# How Have Nutrition Practices in the ICU Changed in the Last Decade (2011-2020): A Scoping Review

**DOI:** 10.7759/cureus.15422

**Published:** 2021-06-03

**Authors:** Subhal B Dixit, Nishant R Tiwari, Kapil G Zirpe, Aditya G Tolat, Khalid I Khatib, Atul P Kulkarni, Yatin Mehta, Rajesh C Mishra, Deepak Govil, Dhruva Chaudhry, Lopa Ahsina Jahan

**Affiliations:** 1 Critical Care Medicine, Sanjeevan Hospital, Pune, IND; 2 Internal Medicine, Byramjee Jeejeebhoy Medical College, Pune, IND; 3 Neurocritical Care, Ruby Hall Clinic, Grant Medical Foundation, Pune, IND; 4 General Surgery, Vardhman Mahavir Medical College and Safdarjung Hospital, New Delhi, IND; 5 Critical Care Medicine, Smt Kashibai Navale Medical College and General Hospital, Pune, IND; 6 Anaesthesiology and Critical Care Medicine, Tata Memorial Hospital, Mumbai, IND; 7 Anaesthesiology and Critical Care Medicine, Medanta-The Medicity, Gurugram, IND; 8 Critical Care Medicine, Sanjivani Super Speciality Hospital, Ahmedabad, IND; 9 Pulmonary and Critical Care Medicine, Pandit Bhagwat Dayal Sharma Post Graduate Institute of Medical Sciences, Rohtak, IND; 10 Critical Care Medicine, MH Samorita Hospital and Medical College, Dhaka, BGD

**Keywords:** medical intensive care unit (micu), nutrition in critical care, icu, early enteral nutrition (een), enteral and parenteral nutrition

## Abstract

Malnutrition is more prevalent in the critically ill than ambulatory patients due to a variety of factors. Strategies employed in the optimization of nutrition practices rely largely on the review of published literature and guidelines. While the last decade was marked by some landmark large randomized controlled trials taking place and some high-quality systematic reviews, it still has left us with many unanswered questions. The evidence generated by these trials can, to a good extent, extrapolate to the developed countries. However, its implementation in developing and third-world countries needs further elaboration and logistical considerations. With this scoping review, we attempt to provide insights into the landmark developments in the decade 2011-2020. Solutions to employ and implement the results of these developments and ways for their corroboration into a larger population are also discussed.

## Introduction and background

Patients in intensive care units (ICU) are often prone to malnourishment because of increased caloric requirements due to metabolic stress and decreased intake due to anorexia [1]. Immobilization, prolonged mechanical ventilation, and profound inflammation levels further compound the situation, making the patients susceptible to skeletal muscle wasting and weakness [2]. Energy deficit in critically ill patients is strongly associated with increased ICU stay, infectious complications, and mortality [3]. Until the beginning of the last decade, the data on nutrition in ICU patients was available as observational and small interventional studies that could not replicate the complexities of interventions in the critically ill. However, with multiple, large, multicentric randomized controlled trials (RCTs) in the last decade - substantial evidence was generated as a byproduct of these trials.

The relationship between malnutrition and adverse outcomes in ICU patients is vastly confounded by age, the severity of disease processes, and other co-existing medical illnesses. In a systematic review, the prevalence of malnutrition in ICU patients ranged from 38% to 78%. This review established an independent association between malnutrition and poorer clinical outcomes [4]. The last decade was marked by some notable developments in technicalities and guidelines in ICU nutrition. In this review, we attempt to provide insights into this progress, focusing on critical changes in the last 10 years.

## Review

Nutritional assessment and requirements

It is tough to define malnutrition in the critically ill. Guidelines recommend that all hospital patients undergo nutritional screening assessment within 48 hours of admission. ICU patients, being at higher risk of malnutrition, frequently require complete nutritional assessment [5]. Anthropometrics and some serum protein markers are not reliable markers of nutrition status and often lead to underdiagnosis of malnutrition [6]. The most effective and all-inclusive ways of full nutrition assessment are Nutrition Risk Screening (NRS) 2002 and Nutrition Risk in the Critically ill (NUTRIC) scoring systems. Both scoring systems pay special attention to nutrition status and disease severity. The nutritional assessment column got particular importance in the Society of Critical Care Medicine (SCCM) and the American Society of Parenteral and Enteral Nutrition (ASPEN) guidelines in 2016. In the year 2011, the concept of NUTRIC score was introduced. It contains six variables (Age, APACHE II [Acute Physiology And Chronic Health Evaluation II], SOFA [Sequential Organ Failure Assessment], # of comorbidities, Days from hospital to ICU admit, IL-6 levels). These are graded 0-1, 0-2, and 0-3 depending on the variable [7].

Traditionally, based on expert opinion, it was recommended that early enteral nutrition (EEN) (<48 hours after admission) is superior to either late enteral nutrition (EN) or parenteral nutrition (PN) [8]. However, due to the lack of high-quality evidence, the strength of the recommendation was low. Evidence generated by an expert committee of the European Society of Intensive Care Medicine (ESCIM) after an extensive literature review provided a recommendation suggesting EEN over late EN or PN [9]. This recommendation was based on the statistically significant decrease in infection rates and a minor, non-significant reduction in mortality with EEN [9].

Critical illnesses are often associated with hypoperfusion, hypoxemia, hypercapnia, and acidosis [1]. Hypoperfusion to the gut decreases motility and hence may hamper the tolerability of enteral nutrition. The ESCIM now recommends holding the EN in case of the patients having uncontrolled hypotension on vasopressors. However, as soon as the patient is relatively stable and has been given vasopressors and intravenous (IV) fluids, it is recommended to provide a trial of low-dose EN [9]. Some might argue that during the critical phase of an illness, when the patient has hypoxia or hypercapnia, feeding the patient might exacerbate the hypercapnia by an increased CO2 generation. Supporting this argument, an RCT including >5,000 patients concluded that late PN in critically ill adults was associated with lower mortality and infection rates than the group where PN was initiated in <48 hours of admission to the ICU [10]. This trial was published in 2006 and was a paradigm-shifting trial that also demonstrated a statistically significant decrease in duration of mechanical ventilation, time of renal replacement therapy, and a mean reduction in healthcare costs in critically ill patients being given late PN [10].

The predictive energy requirements equations like the Harris and Benedict prediction [11] and others [12] have been used conventionally for decades to assess the caloric requirements of critically ill patients. However, inaccuracies ranging up to 60% in these equations led to the introduction of indirect calorimetry (IC) for this assessment [13]. Indirect calorimetry currently serves as a reference standard for assessing the basal metabolic rate and caloric requirements in critically ill adults and children [14]. It uses assumptions that guide the physicians to derive the caloric requirements based on oxygen (O2) consumption and CO2 generation in critically ill patients [14]. Significant differences shown in studies comparing IC to the predictive equations yielded discrepancies ranging from 40% to 60% in estimating the caloric requirements, IC being more accurate, hence became a method of choice for this indication [15].

Choosing the right mode at the right time

Choosing the suitable mode of nutrition in ICU (EN vs. PN) has been a matter of debate for decades. Guidelines of intensive care and nutrition societies worldwide recommend EN over PN unless a clear contraindication to the former exists [16-18]. The practice of EEN was formerly considered a standard of care for most ICU patients [8]. However, the data supporting the evidence was not robust, and hence the recommendation was not strongly supported, albeit it was strongly agreed upon. In recent years, multiple studies that considered the dosage of EN demonstrated a clear benefit in decreased infection rates and hospital stay in patients where EEN was practiced compared to early PN [19-21]. A metanalysis confirmed these findings with a statistically significant difference in infection rates and hospital stay [18]. This data came into light mainly in the last decade and resonated with the earlier thought process of EN over PN in indicated patients.

As mentioned above, the current data suggest higher infection rates with PN than with that of EEN. A systematic review and meta-analysis by Elke et al., looking at the same, reinforced these conclusions [22]. However, most recent studies in this metanalysis found that this was not consistent when a similar dosage of EN and PN was used [22]. The data opened the floodgates to a critical but debatable area of ICU nutrition - ‘hypocaloric nutrition.’ Since then, multiple prospective RCTs got started and completed looking at hypocaloric nutrition. While the matter is still debatable, an inclination towards early hypocaloric nutrition and a shift later to higher EN or PN dosage (depending on the patient profile) seems to be happening (NCT01531335, NCT02577211) [5,18].

Another topic of interest in critically ill patients is the gastric vs. post-pyloric method of enteral nutrition. While a strong consensus recommendation emphasizes the importance of gastric feeding in these patients, lower rates of aspiration and pneumonia in critically ill patients fed via naso-duodenal or nasojejunal route reported in the recent studies also put post-pyloric nutrition ahead in the consideration [19,23-26]. The European Society of Parenteral and Enteral Nutrition (ESPEN) guidelines on ICU nutrition in 2019 recommended post-pyloric nutrition in patients with a high risk of aspiration and patients where the feeding intolerance is not ameliorated with the prokinetic agents [18]. The increased risk of aspiration can be identified by factors such as the patient’s age (age > 70 years), logistical aspects (lower nurse: patient ratio), general patient condition (Impaired consciousness, neurological deficits, poor oral hygiene), and planned interventions (supine positioning, transport out of ICU). However, while interpreting these recommendations, an important factor needs to be considered - the availability of intensive care expertise. Placement of a nasojejunal tube is a procedure practiced by well-trained nurses in advanced critical care medicine, availability of which might be a challenging factor in the ICUs of developing countries [27].

It also leads us into an argument, ‘can we extrapolate the evidence generated in ICU nutrition from developed countries into the developing part of the world like the Indian subcontinent?’ An intriguing review published recently looked at this and derived exciting conclusions [28]. While the quality and strength of evidence generated in developed countries are high, factors like shortage of nurses trained in advanced critical care medicine, lack of advanced equipment in most developing countries render us unable to implement and follow these guidelines strictly. A more wholesome approach, considering the logistical and training factors in the developing countries, is suggested. It can also be achieved by conducting more RCTs in the developing world and applying them to guide clinical practice [29].

Evidence about the idea of initiating PN in patients not tolerating EN or in patients with contraindications to EN has proliferated tremendously in the last decade. While the early institution of PN has been shown to have deleterious effects in terms of increased ICU stay and mechanical ventilation dependency [30], the risks associated with malnutrition in the critically ill are also significant. The heterogeneity in the studies on the timing of starting PN in the critically ill makes doing a metanalysis a daunting task. Extrapolating the data from existing studies to formulate guidelines generally leads to good practice points (GPP); however, the strength of the evidence remains questionable. ESPEN guidelines recommend maximization of efforts to initiate EN before starting PN. At the same time, if EN is contraindicated or intolerable, it is now recommended that PN is started within three to seven days of admission to the ICU [18].

Macronutrients

One of the significant challenges faced during routine critical care practice is the dosage of nutrients given to the patients. A review of ESPEN and ASPEN guidelines from the years 2006 and 2009 yield little guidance on this topic, while the most recent guidelines focus extensively on hypocaloric nutrition, especially in the earlier phase of the critical illness [5,18]. While there are many methods to establish the value of energy expenditure (EE), IC is pretty much the standard of care. When IC is used to assess the EE, hypocaloric nutrition, followed by isocaloric nutrition, is recommended during the early phase of the illness [18]. Also, when the predictive equations are used to assess the EE, ESPEN now recommends using hypocaloric nutrition for the first week of critical illness and isocaloric nutrition from that point onwards [18]. This recommendation has come in accordance with the insulin resistance and hyperglycemia that comes with the critical illness.

The critical illnesses cause proteolysis and muscle protein loss (values ranging up to 1 kg/day), leading to critical illness-induced myopathy complications [31]. For compensating and counteracting these losses, we may have to administer higher dosages of proteins. However, it is imperative to remember that, although proteins and carbohydrates both are macronutrients - their dynamics change in critical illnesses. While we practice hypocaloric nutrition in the acute phase of critical conditions, it is quintessential to administer a higher dose of proteins than we would to a non-critical illness patient, unless, of course, a clear contraindication exists (hepatic/uremic encephalopathy) [18]. Proteins given more than 1.2 g/kg/day have shown survival and morbidity benefit in many studies that happened in the last decade [31-36]. The latest ESPEN and ASPEN guidelines also favor giving 1.3 g/kg/day protein to the patients, even during the early phase of critical illness [5,18]. ESPEN also recommends early restoration of physical activity in critical illnesses to alleviate and counteract the protein loss, but the strength of the recommendation is low [18]. More observational and interventional studies are needed in exercise and physical activity to reach a more definitive conclusion.

While there is a strong consensus about the caloric and protein dosages, the area of dosing for essential fatty acids remains relatively underexplored. Absorption of lipids is impaired in critical illness due to factors such as hypoperfusion [36]. Also, the dynamics of fat metabolism are changed, i.e., higher HDL-cholesterol and lower triglyceride levels in the blood are associated with improved survival in the critically ill [37, 38]. Unsaturated fats given in excess dosages have also been associated with increased incidence of lung injury and immunosuppression. For all these reasons, a glucose/lipids ratio on the lower side is recommended [18,39]. Another question commonly arises about lipids in the nutrition of the critically ill is about the dosage of lipid emulsions in PN formulations. A GPP level and a strong consensus of ESPEN argues in favor of keeping the lipid levels <1.5 g/kg/day in emulsion being administered parenterally to the patient as a part of PN [18]. This intervention also works for keeping the insulin resistance and hyperglycemia of critical illness in check.

The 2006 ESPEN guidelines did not provide a specific and robust recommendation about the dosing of glutamine (GLN) [8]; the latest guidelines recommend GLN in the dose of 0.3 to 0.5 g/kg/day for one-two weeks as soon as EN can be started [18]. Also, barring burns and trauma patients - administration of additional GLN is now not recommended [18].

Micronutrients

Micronutrients are vital and integral parts of any nutritional formulation, and their importance is emphasized even in the guidelines from the last decade (2006 and 2009) [8,40]. Since it would be unethical to conduct an RCT on this area (nutrition with and without micronutrients), the strength of evidence published so far remains low, making it hard to give a strong consensus. ICU patients are more likely to be depleted of micronutrients like zinc and vitamins. Persistently low zinc levels in the blood have also been used as a biomarker for sepsis [41]. ICU patients are also more likely to receive renal replacement therapy [42] and are prone to developing acute and chronic severe micronutrient deficiency [43]. Many studies advocate for giving ‘safe’ doses of antioxidants to critically ill patients to counterbalance the inflammatory oxidative stress associated with these conditions [44,45]. However, their benefits are primarily studied in deficient patients and cannot be extrapolated to the broader population group. The ESPEN 2019 guidelines, thus, recommend the antioxidants like vitamin C only if an evident deficiency exists [18].

Good renal and liver function form bedrocks for maintaining the levels of vitamin D3 in the human body. Critical illnesses are often associated with impairment in either or both, which only adds to the risk encumbered by lack of sunlight exposure in the ICU. Vitamin D deficiency has also been shown to worsen outcomes in excess morbidity, mortality, and ICU stays [46]. For this reason, prolonged ICU stay is associated with vitamin D deficiency which acts as one of the factors in worsening outcomes. The ESPEN now recommends giving a high dose of vitamin D (500,000 UI) within the first week of admission to the ICU in deficient patients [18].

Recommendations in critical medical and surgical illnesses

In hemodynamically unstable critically ill patients, starting with EN becomes a challenge due to bowel ischemia. The ESPEN and ASPEN guidelines from the years 2006 and 2009 also focused on this critical issue; however, the strength of recommendations was affected due to lack of robust evidence [8,41]. The 2019 ESPEN and 2016 ASPEN guidelines with a strong consensus agreement recommend delaying EN in hemodynamically unstable patients, in patients with uncontrolled hypercapnia, acidosis, and hypoxia, and various other medical and surgical illnesses (Overt bowel ischemia, high output intestinal fistula, abdominal compartment syndrome) [5,18]. Restoration towards improvement from this condition should guide the intensivists to institute low dose EN, progressing to isocaloric, and appropriate dose EN. In situations like acute spinal cord injury, open abdomen, severe acute pancreatitis - early introduction of EN is recommended (<48 hours after admission and as soon as the patient is relatively hemodynamically stable) [18].

## Conclusions

This review leads us to an open-ended question: What can we do to improve the ICU nutrition practices further? The answer to this question lies in the grey areas of the existing guidelines. From an overview derived so far, it seems that the guidelines give clear recommendations for lipids in EN and PN, but the consensus remains relatively less strong. Extensive, retrospective studies and reviews of national inpatient databases may help form a more substantial agreement in this case. Telemedicine can lessen the disparities in ICU nutrition practices between well-developed ICUs and ICUs in resource-limited settings. Formulating a strategy for the patients in ICU to tailor their nutrition needs post-discharge upfront may yield better clinical outcomes and lesser prevalence of malnutrition. While large RCTs will be ideal, at times, they may be unethical - retrospective and observational studies are likely to provide us with more ‘real-world’ data. It is of paramount importance that these studies optimize the practices further and work towards eliminating malnutrition in ICU patients.
